# Editorial: 21st International Conference on Bioinformatics (InCoB 2022)—accelerating innovation to meet biological challenges: the role of bioinformatics

**DOI:** 10.3389/fgene.2024.1365223

**Published:** 2024-01-25

**Authors:** Asif M. Khan, Harpreet Singh, Shoba Ranganathan, Takashi Gojobori, Xin Gao

**Affiliations:** ^1^ APBioNET.org, Singapore, Singapore; ^2^ School of Data Sciences, Perdana University, Kuala Lumpur, Malaysia; ^3^ Beykoz Institute of Life Sciences and Biotechnology, Bezmialem Vakif University, Istanbul, Türkiye; ^4^ College of Computing and Information Technology, University of Doha for Science and Technology, Doha, Qatar; ^5^ Hans Raj Mahila Maha Vidyalaya, Jalandhar, India; ^6^ Bioclues.org, Hyderabad, India; ^7^ Applied BioSciences, Macquarie University, Sydney, NSW, Australia; ^8^ National Supercomputing Centre, Singapore, Singapore; ^9^ King Abdullah University of Science and Technology, Thuwal, Saudi Arabia

**Keywords:** bioinformatics and computational biology, knowledge discovery (data mining), biological data analysis, biological databases, data integration, genome informatics, genotype-phenotype relationships, integrative data analysis

The advent of bioinformatics marked a significant paradigm shift in our approach to biological research (Rastogi, 2023). Coined in the 1970s by Dr. Paulien Hogeweg and Ben Hesper, the term initially encapsulated the study of informatic processes in biotic systems. Over the years, the focus of bioinformatics has shifted predominantly to data analysis, often neglecting the comprehensive understanding and computational reproduction of underlying biological processes. Today, bioinformatics stands at the forefront of addressing some of the most daunting challenges in life sciences, redefining our approach to healthcare and scientific research.

A 2017 report by the Biological and Environmental Research Advisory Committee of the U.S. Department of Energy defined the grand challenges in biological systems science for the coming decades (BERAC, 2017). They are briefly outlined as follows:

1) Understand the complexity of plant and microbial metabolism across scales.
2) Develop technologies to identify and engineer metabolic capabilities in various organisms, including bacteria, fungi, archaea, viruses, plants, and communities.
3) Optimise the use of large datasets to integrate omics surveys with biochemical and biophysical measurements.
4) Understand the links between genotype and phenotype in diverse organisms and communities within terrestrial ecosystems.
5) Exploit new and emerging technologies in systems biology and physical measurements to accelerate biological discoveries.

Although the above grand challenges are in relation to energy production and the living environment, they can be generalised by broadening the emphasis to all biological organisms. Research in biological systems aims to comprehensively understand and predict the behaviour of organisms through a combination of genomic science, computational analysis, and experimental methods. Gaining a fundamental understanding of the architecture and operational mechanisms of these systems is crucial for harnessing their natural processes for critical applications. Bioinformatics is a key enabler in deciphering the complexity of biological systems, accelerating innovation in the development of new technologies for metabolic engineering, optimising the use of large-scale biological data, and establishing clear links between genotype, phenotype, and the environment. Its role in the above grand challenges underscores the interdisciplinary nature of modern biological research.

The evolution of the field of life sciences necessitates enhanced bioinformatics education to equip the next-generation of professionals for the dynamic training challenges (Işık et al., 2023). Furthermore, the interdisciplinary nature of bioinformatics projects often leads to challenges in collaboration, particularly due to technical language barriers and differences in work culture among researchers from various backgrounds (Morrison-Smith et al., 2022). These complexities necessitate a holistic approach beyond traditional data analysis and the need to incorporate dynamic modelling, comprehensive education, and training in the field.

This Frontiers Research Topic was created in conjunction with the 21st International Conference on Bioinformatics (InCoB) 2022 (https://incob.apbionet.org/incob22), and it was aligned with the theme of the conference: “Accelerating Innovation to Meet Biological Challenges: The Role of Bioinformatics.” InCoB 2022 was held virtually from 21 to 23 November 2022 across Asia-Pacific and beyond. The conference included keynote talks, presentations of original research results, and poster sessions related to the field of bioinformatics (Hekimoglu et al., 2023).

InCoB (https://incob.apbionet.org), initiated in Bangkok in 2002, has evolved under the auspices of the Asia Pacific Bioinformatics Network (APBioNET; https://www.apbionet.org) into one of Asia’s most prominent bioinformatics conferences. InCoB’s journey through various countries reflects the growing significance and expertise in bioinformatics across the Asia-Pacific region. Hosting the 21st InCoB in collaboration with the Computational Bioscience Research Center (CBRC) at King Abdullah University of Science and Technology (KAUST; https://www.kaust.edu.sa) marked a significant milestone, bringing the conference to the Middle East for the first time. This virtual event demonstrated the resilience and commitment of the bioinformatics community to maintain continuity and global connection amidst challenging times, such as the COVID-19 pandemic. The untimely demise of the founding Director of CBRC, Dr. Vladimir Bajic in 2019, also resulted in delays in plans to host the conference. Dr. Bajic, a world-renowned pioneer in bioinformatics, made immense contributions to science and the broader community (Zhang et al., 2019), and he will be missed.

KAUST, since its inception in 2009, has rapidly established itself as a crucible of scientific inquiry and innovation. With its focus on addressing some of the most pressing global challenges in health, water, energy, and the environment, KAUST represents a microcosm of the scientific diversity and interdisciplinary collaboration that is crucial for innovation in bioinformatics and computational biology (https://cemse.kaust.edu.sa/cbrc). The CBRC, established at KAUST’s inception in 2009, advances research by integrating computer science, artificial intelligence, and mathematical modeling in the areas of life sciences, human health, and environmental studies.

APBioNET has been a driving force in promoting and developing bioinformatics in the Asia-Pacific region since 1998 (Khan et al., 2013). Its mission encompasses a spectrum of activities, including training, education, infrastructure development, and research in bioinformatics. APBioNET’s role transcends geographical boundaries, facilitating technical coordination and collaboration with international scientific bodies. The organisation’s initiatives have significantly contributed to elevating bioinformatics, an indispensable tool in modern biological research, and positioning itself as a key player in addressing global scientific challenges.

The Research Topic collection received 13 submissions. Covering the various facets of the theme, below we summarise the five submissions published as part of this collection.

The emergence of antimicrobial resistance (AMR) has been identified as a critical threat to global health, with the World Health Organisation (WHO) reporting a continuous rise in the resistance of bacterial pathogens to antibiotics (WHO, 2022). Yang et al. addressed the pressing need for advanced methods in predicting the Minimum Inhibitory Concentrations (MIC) of antibiotics, a critical factor in effectively treating bacterial infections. Leveraging machine learning on the *Salmonella enterica* pan-genome, the authors introduced a novel approach to predict MIC values, potentially improving the way we understand and tackle AMR. Their method of conducting feature selection across a gene set, rather than restricting to a small portion of known AMR genes, not only improves the prediction of antibiotic MIC but also paves the way for identifying novel genes linked to AMR.

Gastric cancer remains one of the leading causes of cancer mortality worldwide, with a significant need for new therapeutic strategies (Ferlay et al., 2020). The integration of traditional medicine and modern cancer treatment offers a promising avenue for novel therapeutic approaches. Li et al. through transcriptomic analysis of gastric tumour tissues, identified immune-related differentially expressed genes and analysed inhibitory binding, with validation, of two select essential genes (TLR4 and KRAS) to 12 herbal medicines and 27 herbal ingredients for their potential in treating gastric cancer, modulating the tumour microenvironment and ferroptosis in cancer cells. The work represents an intersection of traditional medicinal knowledge and contemporary bioinformatics, showcasing the potential of holistic approaches in medical research.

Diffuse Large B-cell Lymphoma (DLBCL), the most common type of non-Hodgkin lymphoma, presents significant challenges in treatment due to its genetic heterogeneity (SEER, 2023). A better understanding of its molecular underpinnings is crucial for improving patient outcomes. Wu et al. introduced a novel prognostic model based on microtubule-associated genes (MAGs) mRNA expression using clinical data from DLBCL patients, enhancing prognosis predictions and potentially guiding targeted therapies. It emphasises the role of bioinformatics in unravelling the complexities of genetic diseases and tailoring patient-specific treatments.

Tuberculosis, caused by *Mycobacterium tuberculosis*, remains a global health burden, with increasing drug resistance complicating treatment efforts (WHO, 2021). Understanding the pathogen’s genetic regulation is vital to developing effective interventions. Dey et al. offers novel insights into the DNA binding sites of 21 transcription factors in *M. tuberculosis*, revealing how structural properties of DNA influence the pathogenicity and drug resistance of the pathogen. This work exemplifies the critical role of bioinformatics in infectious disease research, offering new perspectives and methodologies in the effort against one of the world’s most enduring pathogens.

The advent of third-generation sequencing technologies has been transformative for genomics research, yet the high error rate of long-read sequencing poses challenges, particularly in accurately representing isoform diversity (Weirather et al., 2017). Addressing this challenge, Zhu and Liao introduce LCAT (long-read error correction algorithm for transcriptome sequencing data), a wrapper algorithm for MECAT (method for self-error correction and genome assembly with third-generation sequencing reads) to enhance the accuracy of long-read sequencing while preserving isoform diversity. LCAT represents a significant contribution to genomics, demonstrating how bioinformatics can provide tools to refine and improve our understanding of genetic complexity across diverse biological systems.

The array of studies presented at InCoB 2022, ranging from antimicrobial resistance to better cancer prognosis predictions and targeted therapies and novel insights into pathogen genetics, highlights the transformative role of bioinformatics in contemporary biology. These research works address immediate challenges in biology and medicine and pave the way for future explorations, offering new methodologies and insights. InCoB 2022, through its diverse array of presentations and discussions, has reinforced the essential role of bioinformatics as a critical player in the global effort to address biological challenges. The tradition of publishing high-quality bioinformatics research in Frontiers in Genetics was continued at InCoB 2023, Brisbane, Australia (https://www.frontiersin.org/research-topics/57333).
